# Corrigendum

**DOI:** 10.2478/aiht-2023-74-3704corr

**Published:** 2023-12-29

**Authors:** Angel Dzhambov

In the Statistical analysis subsection of the article **Dzhambov AM, Dikova K, Georgieva T, Panev TI, Mukhtarov P, Dimitrova R. Short-term effects of air pollution on hospital admissions for cardiovascular diseases and diabetes mellitus in Sofia, Bulgaria (2009–2018)** published in *Archives of Industrial Hygiene and Toxicology* 2023;74(1):48–60 (doi: 10.2478/aiht-2023-74-3704), we wrote the following:

“Pollutant concentrations, except for CO, were re-scaled so that one-unit increase corresponded to 10 μg/m^3^, while CO was left in its original scale of 1 mg/m^3^.”

However, upon publication, we realised that we had inadvertently rescaled all pollutant concentrations so that one-unit increase corresponded to 10 mg/m^3^ of CO. This means that the coefficients (incidence rate ratios; IRR) associated with the lagged effects of CO on hospital admissions for “lags 0–7” and “net 0–7 days”, given at the bottom right panel for CO in Figures 3–8, represent a change in the IRR of hospital admission for every 10 mg/m^3^ increase in CO, even though we have referred to them as IRR per 1 mg/m^3^ increase in CO. While this does not invalidate the coefficients, the wrong interpretation we originally provided in our article is responsible for the seemingly exaggerated effects of CO, which we observed in the lagged models and commented on as suspicious. For a correct interpretation of these coefficients, the reader should either interpret them as IRR of hospital admission per 10 mg/m^3^ increase in CO or divide them by 10 so that they reflect IRR of hospital admission per 1 mg/m^3^ increase in CO.

## Editor's note on the corrigendum

Considering the nature of the authors’ error, we will not replace the online article on our Sciendo platform and HRČAK repository but invite the readers to interpret the coefficients as instructed by authors.

*Nevenka Kopjar, Editor in Chief*

